# P2X7R blockade prevents NLRP3 inflammasome activation and brain injury in a rat model of intracerebral hemorrhage: involvement of peroxynitrite

**DOI:** 10.1186/s12974-015-0409-2

**Published:** 2015-10-17

**Authors:** Liang Feng, Yizhao Chen, Rui Ding, Zhenghao Fu, Shuo Yang, Xinqing Deng, Jun Zeng

**Affiliations:** The National Key Clinical Specialty, The Engineering Technology Research Center of Education Ministry of China, Guangdong Provincial Key Laboratory on Brain Function Repair and Regeneration, Department of Neurosurgery, Zhujiang Hospital, Southern Medical University, Guangzhou, 510282 China; Department of Neurosurgery, Jingmen No. 1 People’s Hospital, Jingmen, 448000 Hubei China; Department of Neurosurgery, The Fifth Affiliated Hospital of Southern Medical University, Guangzhou, 510900 China; Department of Neurosurgery, Gaoqing Campus of Central Hospital of Zibo, Gaoqing People’s Hospital, Gaoqing, Zibo, 256300 Shandong China; Department of Neurosurgery, 999 Brain Hospital, Jinan University, Guangzhou, 510510 Guangdong China

**Keywords:** P2X7R, NLRP3, Peroxynitrite, Intracerebral hemorrhage, NOX2, IL-1β

## Abstract

**Background:**

The NLR family, pyrin domain-containing 3 (NLRP3) inflammasome plays a key role in intracerebral hemorrhage (ICH)-induced inflammatory injury, and the purinergic 2X7 receptor (P2X7R) is upstream of NLRP3 activation. This study aimed to investigate how P2X7R functions in ICH-induced inflammatory injury and how the receptor interacts with the NLRP3 inflammasome.

**Methods:**

Rats were treated with P2X7R small interfering RNA (siRNA) 24 h before undergoing collagenase-induced ICH. A selective P2X7R inhibitor (blue brilliant G, BBG) or a peroxynitrite (ONOO^−^) decomposition catalyst (5,10,15,20-tetrakis(4-sulfonatophenyl)porphyrinato iron(III) [FeTPPS]) was injected 30 min after ICH. Brain water content, hemorrhagic lesion volume, and neurological deficits were evaluated, and western blot, immunofluorescence, and terminal deoxynucleotidyl transferase dUTP nick end labeling (TUNEL) were carried out.

**Results:**

Striatal P2X7R and NLRP3 inflammasomes were activated after ICH. Gene silencing of P2X7R suppressed NLRP3 inflammasome activation and interleukin (IL)-1β/IL-18 release and significantly ameliorated brain edema and neurological deficits. Additionally, enhanced NADPH oxidase 2 (NOX2, gp91^phox^) and inducible nitric oxide synthase (iNOS), as well as their cytotoxic product (ONOO^−^) were markedly attenuated by BBG treatment following ICH. This was accompanied by downregulations of the inflammasome components, IL-1β/IL-18 and myeloperoxidase (MPO, a neutrophil marker). Most importantly, inflammasome activation and IL-1β/IL-18 release were significantly inhibited by ONOO^−^ decomposition with FeTPPS.

**Conclusions:**

Our findings implicate that P2X7R exacerbated inflammatory progression and brain damage in ICH rats possibly via NLRP3 inflammasome-dependent IL-1β/IL-18 release and neutrophil infiltration. ONOO^−^, a potential downstream signaling molecule of P2X7R, may play a critical role in triggering NLRP3 inflammasome activation.

**Electronic supplementary material:**

The online version of this article (doi:10.1186/s12974-015-0409-2) contains supplementary material, which is available to authorized users.

## Background

Spontaneous intracerebral hemorrhage (ICH) is a devastating stroke subtype, with high morbidity and mortality [1]. Unfortunately, no satisfactory pharmacologic treatments have been found for clinical practice, mainly due to a lack of knowledge underlying the mechanisms of post-ICH brain damage. There is an urgent need to clarify the pathophysiology of this disease to identify effective therapies.

Accumulating evidence suggests that innate immunity and inflammatory responses are involved in ICH-induced secondary brain injury [1–3]. The intracellular Nod-like receptors have recently been shown to play a critical role in the process of innate immunity and inflammatory responses [4]. The NLR family, pyrin domain-containing 3 (NLRP3) inflammasome, the best characterized member of Nod-like receptor family, is a multiprotein complex that contains the adaptor protein apoptosis-associated speck-like protein containing a CARD (ASC) and the effector caspase-1. Once activated, caspase-1 can cleave the pro forms of interleukin (IL)-1β and IL-18 into their mature and active forms, which leads to the recruitment and activation of other immune cells, such as neutrophils [5]. In this regard, evidence indicates that the NLRP3 inflammasome plays a pivotal role in ICH [2] and other central nervous system (CNS) conditions [6–11], but the precise mechanisms associated with inflammasome activation continue to be debated.

The role of ATP-gated transmembrane cation channel P2X7R in the signaling cascade has received particular attention due to its widespread involvement as a key regulatory element of NLRP3 inflammasome activation [12]. A growing number of studies have demonstrated the important pathophysiological functions of P2X7R in CNS disorders, including ischemic stroke, subarachnoid hemorrhage, neurotrauma, epilepsy, neuropathic pain, and neurodegenerative illnesses [13, 14]. However, the specific role of P2X7R in ICH has not yet been established, and the interaction between P2X7R and the NLRP3 inflammasome in the development of ICH-induced brain injury remains unclear.

In the case of ICH, both NADPH oxidase 2 (NOX2) and inducible nitric oxide synthase (iNOS) have been reported to contribute to brain injury; knockout mice exhibit less brain edema and cell death than wild-type controls following ICH [15, 16]. Superoxide anion (O_2_^−^) and nitric oxide (NO), released through NOX and iNOS in activated microglia, act as devastating pro-inflammatory mediators in CNS diseases [17]. More importantly, peroxynitrite (ONOO^−^), the product of a diffusion-controlled reaction of NO with O_2_^−^, is a more potent oxidant species and is involved in the pathologies of ischemic stroke, neurotrauma, and neurodegenerative diseases [18–20]. We previously demonstrated that abundant ONOO^−^ formed in a hemoglobin (Hb)-induced ICH rat model [21], but the exact mechanisms of ONOO^−^ in brain injury after ICH have not been fully characterized. Besides its ability to oxidize or nitrate proteins, lipids, and DNA, ONOO^−^ can also lead to destructive pathological consequences by triggering the activation of several biochemical pathways engaged in the development of neuroinflammation and IL-1β production [22–24]. However, the precise link between ONOO^−^ formation and IL-1β secretion in ICH is unclear.

The P2X7R acts as an upstream molecule of NOX2 activation signaling in many in vivo and in vitro disease models [25–27]. NOX2-mediated oxidative stress was recently proposed to be responsible for activation of the NLRP3 inflammasome and subsequent neurovascular damage in ischemic stroke [7]. Notably, P2X7R-dependent NADPH oxidase activation and ONOO^−^ formation play key roles in caspase-1 and IL-1β processing in endotoxin-primed human monocytes [28]. In an animal model of lipopolysaccharide (LPS)-induced striatum injury, activated P2X7R in microglia was associated with increased iNOS and 3-nitrotyrosine (3-NT, a reliable marker of ONOO^−^), and this was reversed by the P2X7R antagonist oxidized ATP (oxATP) [29]. Despite this knowledge, the potential roles of P2X7R and NLRP3 inflammasomes and NOX2/iNOS-dependent ONOO^−^ formation in the development of ICH-induced brain damage remain to be clarified.

We hypothesized that ONOO^−^, formed from NOX2-derived O_2_^−^ and iNOS-derived NO, may be involved in transducing P2X7R-mediated IL-1β/IL-18 production and brain injury via NLRP3 inflammasome activation after ICH. We first investigated the expression profiles of P2X7R and the NLRP3 inflammasome components. Next, a mixed small interfering (si) RNA was applied to knock down P2X7R in vivo, and alterations in NLRP3 inflammasome components and functional outcomes were measured. We then explored the therapeutic effect of the selective P2X7R antagonist, blue brilliant G (BBG). Additionally, we observed iNOS- and NOX2-dependent formation of ONOO^−^ and their alterations in ICH rats following BBG treatment. Finally, to determine whether ONOO^−^ is involved in P2X7R-mediated NLRP3 inflammasome activation, we used an ONOO^−^ decomposition catalyst in vivo and measured the expression levels of P2X7R and NLRP3 inflammasome components.

## Materials and methods

### Animals

Sprague–Dawley (SD) male rats weighing 280–320 g were purchased from the Animal Experiment Center of Southern Medical University (Guangzhou, China). All experimental procedures and animal care were approved by the Southern Medical University Ethics Committee.

### ICH model

After anesthetization (0.3 ml/100 g, 10 % chloral hydrate, Sigma-Aldrich, St. Louis, MO, USA), an incision was made on the skin along the sagittal midline to expose the skull. A burr hole (1 mm) was drilled 3 mm lateral and 1 mm anterior to the bregma, then a 30-gauge needle was inserted through the burr hole into the striatum (6 mm ventral from the skull surface), and ICH was induced by stereotaxic infusion of bacterial collagenase VII-S (0.25U in 1.0 μl sterile saline, Sigma-Aldrich) over a 10-min period. In the Sham group, rats were subjected to only a needle insertion as described above. The needle was kept in situ for another 10 min to prevent backflow and then slowly removed. The craniotomies were sealed with bone wax. Rats were allowed to recover in separate cages with free access to food and water.

### Experimental protocol

Four separate experiments were conducted as shown in Additional file 1.

#### Experiment 1

Thirty-six rats were divided into six groups (Sham, and 6, 12, 24, 48, and 72 h after ICH). The expression levels of P2X7R, NLRP3, ASC, and caspase-1 were detected by western blot. The tissue for immunofluorescence (IF) was collected 24 h after ICH induction.

#### Experiment 2

Eighty-eight rats were randomized into four groups: Sham, Vehicle (ICH + saline, intracerebroventricular injection), Scramble small interfering RNA (siRNA) (1000 pmol, 2 μl, ICH + scramble siRNA), and P2X7R siRNA (1000 pmol, 2 μl, ICH + P2X7R siRNA). siRNA silencing efficacy was assessed by western blot. Brain water content and modified Neurological Severity Score (mNSS) were also measured.

#### Experiment 3

One hundred and thirty-two rats were randomized into four groups: Sham, Vehicle (ICH + saline, intraperitoneal injection), BBG (50 mg/kg), and BBG (100 mg/kg). For the 72-h study, BBG was administered daily by intraperitoneal injection. Western blot, hematoxylin and eosin (H&E) staining, immunofluorescence (IF), and terminal deoxynucleotidyl transferase dUTP nick end labeling (TUNEL) were measured 24 h after ICH induction; mNSS and brain water content were detected at both 24 and 72 h.

#### Experiment 4

Thirty-three rats were randomized into three groups: Sham, Vehicle (ICH + saline, intraperitoneal injection), and FeTPPS (30 mg/kg). Western blotting was performed 24 h after ICH induction.

### siRNA and drug delivery

BBG (Sigma-Aldrich) was diluted at 50 and 100 mg/kg in Vehicle (saline) solution. Rats were treated intraperitoneally with either BBG or Vehicle immediately after ICH induction and at 12, 36, and 60 h.

For in vivo siRNA administration, P2X7R siRNA or non-silencing RNA (Sigma-Aldrich) was applied 24 h before ICH by intracerebroventricular injection as previously described [2]. A cranial burr hole (1 mm) was drilled, and a 30-gauge needle was inserted stereotaxically into the right lateral ventricle. To improve the gene silence efficiency, two different sequences targeting P2X7R siRNA were combined (a)sense:5′-CAGUGAAUGAGUACUACUA-3′; antisense:5′-UAGUAGUACUCAUUCACUG-3′; (b)sense:5′-CUCUUGAGGAGCGCCGAAA-3′;antisense:5′-UUUCGGCGCUCCUCAAGAG-3′. siRNA was dissolved in RNA free water. Scrambled control siRNA (1000 pmol, 2 μl), P2X7R siRNA (1000 pmol, 2 μl), or RNA free water (2 μl) was delivered intracerebroventricularly for 2 min. The needle was left in place for an additional 10 min after injection and then slowly withdrawn.

FeTPPS (Millipore, Billerica, MA, USA) was diluted to 30 mg/kg in Vehicle (saline) solution. Rats were treated intraperitoneally with either FeTPPS or Vehicle immediately and at 12 h after ICH induction. The FeTPPS dose was selected based on our previous reports that showed that it is effectively protected against injury [30].

### RT-PCR

Rats were anesthetized and decapitated. Lesioned tissues (about 40 mg) were obtained, and total RNA was extracted from the tissue with GeneJET™ RNA Purification Kit (Thermo Fisher Scientific Inc., Waltham, MA, USA). RNA (1 μg) was reverse-transcribed to cDNA with high capacity (Life Technologies, Carlsbad, CA, USA). RT-PCR was performed in an ABI Prism 7500 sequence detection system (Applied Biosystems, Foster City, CA, USA) using specific primers designed from known sequences. GAPDH was used as an endogenous control gene. Sequence-specific primers for P2X7R, NLRP3, and GADPH were as follows:

P2X7R, 5′-CTACTCTTCGGTGGGGGCTT-3′

(forward primer),

P2X7R, 5′-CTCTGGATCCGGGTGACTTT-3′

(reverse primer);

NLRP3, 5′-CTGCATGCCGTATCTGGTTG-3′

(forward primer),

NLRP3, 5′-GCTGAGCAAGCTAAAGGCTTC-3′

(reverse primer);

GAPDH, 5′-AGACAGCCGCATCTTCTTGT-3′

(forward primer),

GAPDH, 5′- TGATGGCAACAATGTCCACT-3’

(reverse primer);

### Western blot

Western blotting was performed as described previously [30]. The following primary antibodies were used: rabbit polyclonal anti-P2X7R (1:1000, Alomone Labs, Jerusalem, Israel), rabbit polyclonal anti-NLRP3 antibody (1:1000, Santa Cruz, Biotechnology, Santa Cruz, CA, USA), rabbit polyclonal anti-ASC antibody (1:500, Abclonal, Cambridge, MA, USA), mouse monoclonal anti-caspase-1 p20 antibody (1:1000, Santa Cruz Biotechnology), rabbit polyclonal anti-IL-1β antibody (1:1000, Millipore), rabbit monoclonal anti-IL-18 antibody (1:1000, Abcam, Cambridge, UK), mouse monoclonal anti-nitrotyrosine antibody (1:1000, Abcam), mouse monoclonal anti-iNOS antibody (1:200, Santa Cruz Biotechnology), mouse monoclonal anti-gp91^phox^ antibody (1:2000, BD Transduction Laboratories, San Jose, CA, USA), and rabbit polyclonal anti-myeloperoxidase antibody (MPO, 1:500, Abcam). GAPDH (1:1000, Cell Signaling Technology, Danvers, MA, USA) was employed as the loading control. Blot bands were quantified by densitometry with ImageJ software (National Institutes of Health, Baltimore, MD, USA).

### Paraffin section preparations

The sections were processed as previously described [30] with minor modifications. After anesthetization, rats were transcardially perfused with 200 ml saline followed by 400 ml 4 % paraformaldehyde solution. Brain tissues were then removed and fixed by immersion in the same solution at 4 °C for 24 h. After dehydration and vitrification, they were embedded in paraffin, and 3-μm sections were prepared. Sections were dewaxed, rehydrated, and then processed for IF and TUNEL.

### Histological examination

Coronal sections (1 mm apart) [31] were prepared accordingly and then stained with H&E. Hemorrhagic volumes were calculated using Image Pro Plus 6.0 software (Media Cybernetics, USA) to span the entire hematoma [32].

### IF

Antigen retrieval was performed by heat treatment in a microwave oven for 21 min in Tris–EDTA buffer solution (0.05 mol/L Tris, 0.001 mol/L EDTA; pH 8.5). Sections were incubated for 30 min in 5 % bovine serum albumin (BSA) and then incubated at 4 °C overnight with primary antibodies (rabbit polyclonal anti-P2X7R, 1:500, Alomone labs; mouse monoclonal anti-3-Nitrotyrosine, 1:400, Abcam; rabbit polyclonal anti-nitrotyrosine antibody, 1:200, Millipore; rabbit polyclonal anti-MPO antibody, 1:50, Abcam; rabbit polyclonal anti-iNOS antibody, 1:40, Santa Cruz Biotechnology; rabbit polyclonal anti-Iba-1 antibody, 1:600, WAKO, Osaka, Japan; goat polyclonal anti-Iba-1 antibody, 1:300, Abcam; mouse monoclonal gp91^phox^ antibody, 1:400, BD Transduction Laboratories). For double-staining experiments, primary antibodies were separately incubated overnight at 4 °C. After they were washed with phosphate-buffered saline (PBS), sections were then incubated with secondary antibodies. Images were obtained using confocal microscopes (FV10i-W, Olympus, Tokyo, Japan; LSM780, Zeiss, Oberkochen, Germany).

### TUNEL

At 24 h after ICH, TUNEL staining was performed with an in situ apoptosis detection kit (Roche, Basel, Switzerland) according to the manufacturer’s instruction. For NeuN and TUNEL co-staining, the sections were first labeled with a NeuN antibody (1:400, Abcam), followed by TUNEL. The slides were analyzed using a fluorescence microscope (Bx51, Olympus).

### Brain water content measurement

Brain edema was evaluated by a common wet/dry method as previously described [33]. Briefly, at 24 or 72 h post-ICH, rats were anesthetized and decapitated. The brains were removed and immediately separated into contralateral and ipsilateral hemispheres and the cerebellum and wet weighed. The cerebellum was used as an internal control. Brain specimens were dried in an oven at 100 °C for 24 h to obtain the dry weight. The water content was expressed as a percentage of the wet weight: ([wet weight] – [dry weight]) / (wet weight) × 100 %.

### Behavioral testing

Behavioral tests were assessed with mNSS at 24 and 72 h after ICH by an investigator who was blinded to the experimental groups [34].

### Statistical analysis

Data are shown as mean ± SD. Statistical analysis was performed using SPSS 13.0 (SPSS Inc., Chicago, IL, USA). Comparison between groups was determined by Student’s *t* tests or one-way analysis of variance (ANOVA) followed by least significant difference (LSD) tests with multiple comparisons. The statistically significant level was *P* < 0.05.

## Results

### P2X7R was increased and mainly expressed in microglia cells following ICH

**Fig. 1:**
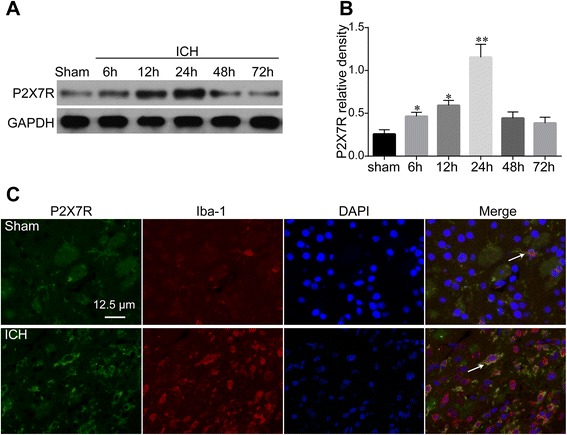
Expression profile of P2X7R and its cellular location after collagenase-induced intracerebral hemorrhage (ICH). Western blot analysis (**a**) for the time course of P2X7R expression in the ipsilateral hemisphere of Sham and ICH rats within 72 h. Quantification of P2X7R (**b**) expression is shown, *n* = 4 rats per group and time point. Confocal images (**c**) of double immunofluorescence for P2X7R expression in Iba-1-positive microglia 24 h following ICH, *n* = 6 rats per group. Scale bar = 12.5 μm. Data represent means ± SD. ^*^ *P* < 0.05 vs. Sham, ^**^ *P* < 0.01 vs. Sham. *GAPDH* glyceraldehyde 3-phosphate dehydrogenase, *ICH* intracerebral hemorrhage

Protein content was analyzed at different time points after injection to investigate whether P2X7R would respond to collagenase-induced ICH. As shown by western blot (Fig. 1a, b), P2X7R levels were significantly elevated at 6 h after ICH (*P* < 0.05 vs. Sham) and peaked at around 24 h (*P* < 0.01 vs. Sham) when P2X7R levels were nearly 4.5 times more than those in the Sham group. Following the peak, P2X7R levels decreased, returning close to baseline levels at 72 h.

Double immunolabeling was performed to identify the cell type that expresses P2X7R. The results showed that P2X7R was predominantly expressed in microglia cells (Fig. 1c) and not in other cell types, such as astrocytes or neurons (Additional file 2).

### NLRP3, ASC, and caspase-1 were upregulated after ICH

**Fig. 2:**
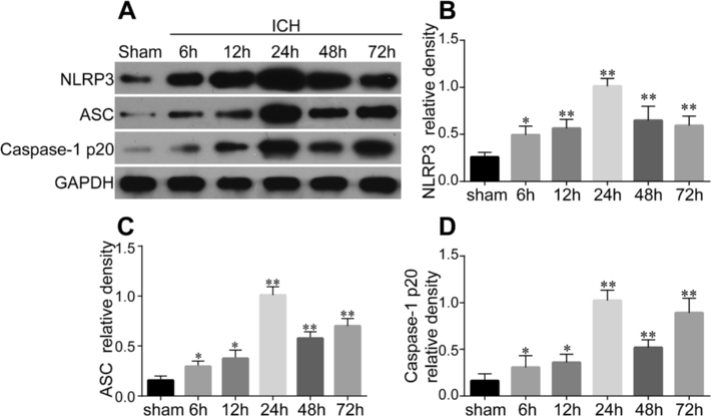
Expression profiles of NLRP3, ASC, and caspase-1 p20 subunit after ICH. Western blot analysis (**a**) for the time course expressions of NLRP3 (**b**), ASC (**c**), and caspase-1 p20 subunit (**d**) in the ipsilateral hemisphere of Sham and ICH rats within 72 h. Quantification of NLRP3, ASC, and caspase-1 p20 subunit expression is shown, respectively, *n* = 4 rats per group and time point. Data represent means ± SD. ^*^ *P* < 0.05 vs. Sham, ^**^ *P* < 0.01 vs. Sham. *ASC* adaptor protein apoptosis-associated speck-like protein containing a CARD, *GAPDH* glyceraldehyde 3-phosphate dehydrogenase, *ICH* intracerebral hemorrhage, *NLRP3* pyrin domain-containing 3

NLRP3 inflammasome has been proposed to be downstream of P2X7R [12]. We evaluated the expressions of NLRP3 inflammasome components by western blot (Fig. 2a). NLRP3 (Fig. 2b), ASC (Fig. 2c), and cleaved caspase-1 (Fig. 2d) were significantly upregulated at 6 h (*P* < 0.05 vs. Sham) and reached their peak at 24 h post-ICH (*P* < 0.01 vs. Sham). Following this peak, levels of all three proteins declined but still remained higher than those in the Sham group at 48 h (*P* < 0.05 vs. Sham) and 72 h (*P* < 0.05 vs. Sham).

### P2X7R RNA interference reduced brain water content and improved neurological outcomes

**Fig. 3:**
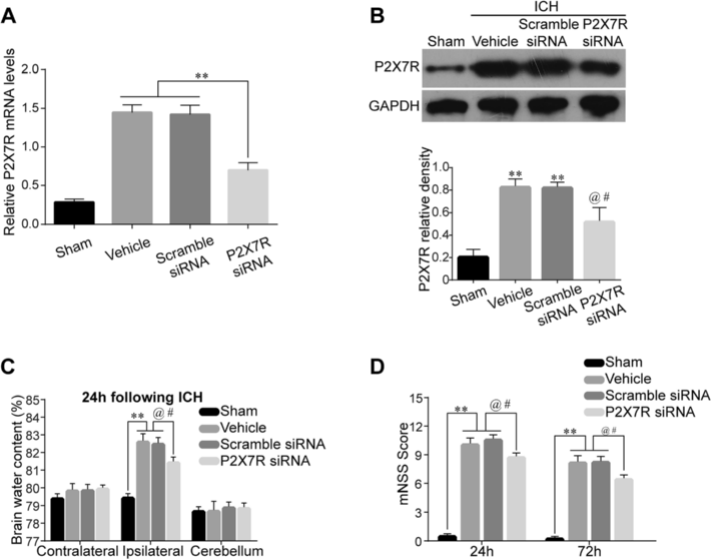
Effects of P2X7R small interfering RNA (siRNA) treatment in ICH rats. RT-PCR of P2X7R after siRNA treatment 24 h following ICH, *n* = 6 rats per group (**a**). Western blot assay and quantification of P2X7R (**b**) after siRNA treatment 24 h following ICH, *n* = 4 rats per group. Brain edema (**c**) at 24 h after ICH, *n* = 6 rats per group. mNSS (**d**) at 24 and 72 h after ICH, *n* = 6 rats per group. Data represent means ± SD. ^**^ *P* < 0.01 vs. Sham, ^*@*^ *P* < 0.05 vs. Vehicle, ^*#*^ *P* < 0.05 vs. ICH + Scramble siRNA. *GAPDH* glyceraldehyde 3-phosphate dehydrogenase, *mNSS* modified Neurological Severity Score, *siRNA* small interfering RNA

We next explored whether P2X7R is involved in brain injury following ICH. Two P2X7R siRNA mixtures were applied 24 h before ICH induction. Silencing efficacy by RT-PCR demonstrated a significant inhibitory effect of P2X7R siRNA on its mRNA levels (*P* < 0.01) (Fig. 3a). Western blot (Fig. 3b) showed 41.3 and 40.7 % reductions of P2X7R in the P2X7R siRNA group compared with the Vehicle and Scramble siRNA groups, respectively (both *P* < 0.05), at 24 h after ICH. Brain water content in the ipsilateral hemisphere was significantly increased in the Vehicle (82.56 ± 0.72 % vs. Sham, 79.40 ± 0.44 %, *P* < 0.01) and Scramble siRNA (82.44 ± 0.75 % vs. Sham, 79.40 ± 0.44 %, *P* < 0.01) groups at 24 h post-ICH, while that in P2X7R siRNA group was decreased to 81.39 ± 0.58 % (*P* < 0.05 vs. Vehicle or Scramble siRNA) (Fig. 3c). Consistent with the brain edema results, P2X7R siRNA administration significantly ameliorated neurological deficits at 24 h (8.66 ± 1.15 vs. Vehicle, 10.00 ± 1.95, *P* < 0.05; vs. Scramble siRNA, 10.50 ± 1.26, *P* < 0.05) and 72 h (6.33 ± 0.81 vs. Vehicle, 8.10 ± 1.96, *P* < 0.05; vs. Scramble siRNA, 8.16 ± 1.16, *P* < 0.05) post-ICH (Fig. 3d).

### P2X7R RNA interference inhibited NLRP3 inflammasome activation and subsequent IL-1β/IL-18 release

**Fig. 4:**
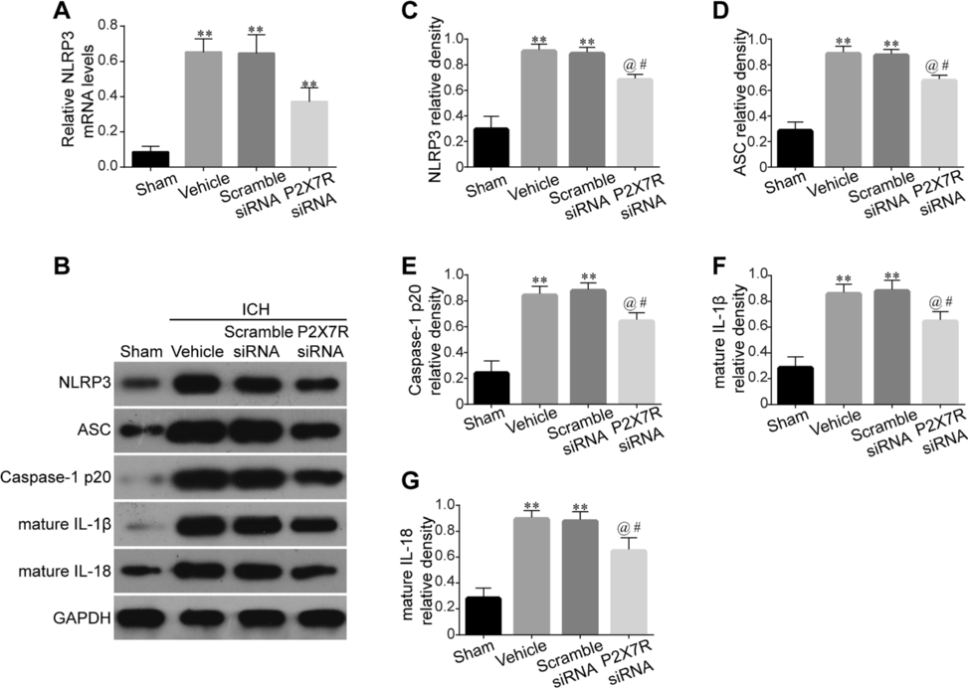
Effects of P2X7R siRNA on NLRP3 inflammasome activation and IL-1β/IL-18 maturation after ICH. RT-PCR of NLRP3 after P2X7R siRNA treatment 24 h following ICH (**a**), *n* = 6 rats per group. Western blot assay (**b**) and quantification of NLRP3 (**c**), ASC (**d**), caspase-1 p20 subunit (**e**), IL-1β (**f**), and IL-18 (**g**) after P2X7R siRNA treatment 24 h following ICH, *n* = 4 rats per group. Data represent means ± SD. ^**^ *P* < 0.01 vs. Sham, ^*@*^ *P* < 0.05 vs. Vehicle, ^*#*^ *P* < 0.05 vs. ICH + Scramble siRNA. *ASC* adaptor protein apoptosis-associated speck-like protein containing a CARD, *GAPDH* glyceraldehyde 3-phosphate dehydrogenase, *ICH* intracerebral hemorrhage, *IL* interleukin, *NLRP3* The NLR family, pyrin domain-containing 3

NLRP3 inflammasome is actively involved in brain injury after ICH [2]. We further clarify the role of P2X7R in NLRP3/ASC/caspase-1 activation and the subsequent processing of IL-1β/IL-18. P2X7R siRNA treatment significantly reduced NLRP3 mRNA expression (*P* < 0.01) (Fig. 4a). The protein levels of NLRP3 inflammasome component and IL-1β/IL-18 production were evidently elevated in the Vehicle and Scramble siRNA groups at 24 h after ICH (*P* < 0.01). P2X7R siRNA treatment significantly suppressed caspase-1 activation and the subsequent secretion of mature IL-1β/IL-18 (*P* < 0.05) (Fig. 4b–f).

### BBG deceased post-ICH neurological deficits, brain water content, and neuronal apoptosis

**Fig. 5:**
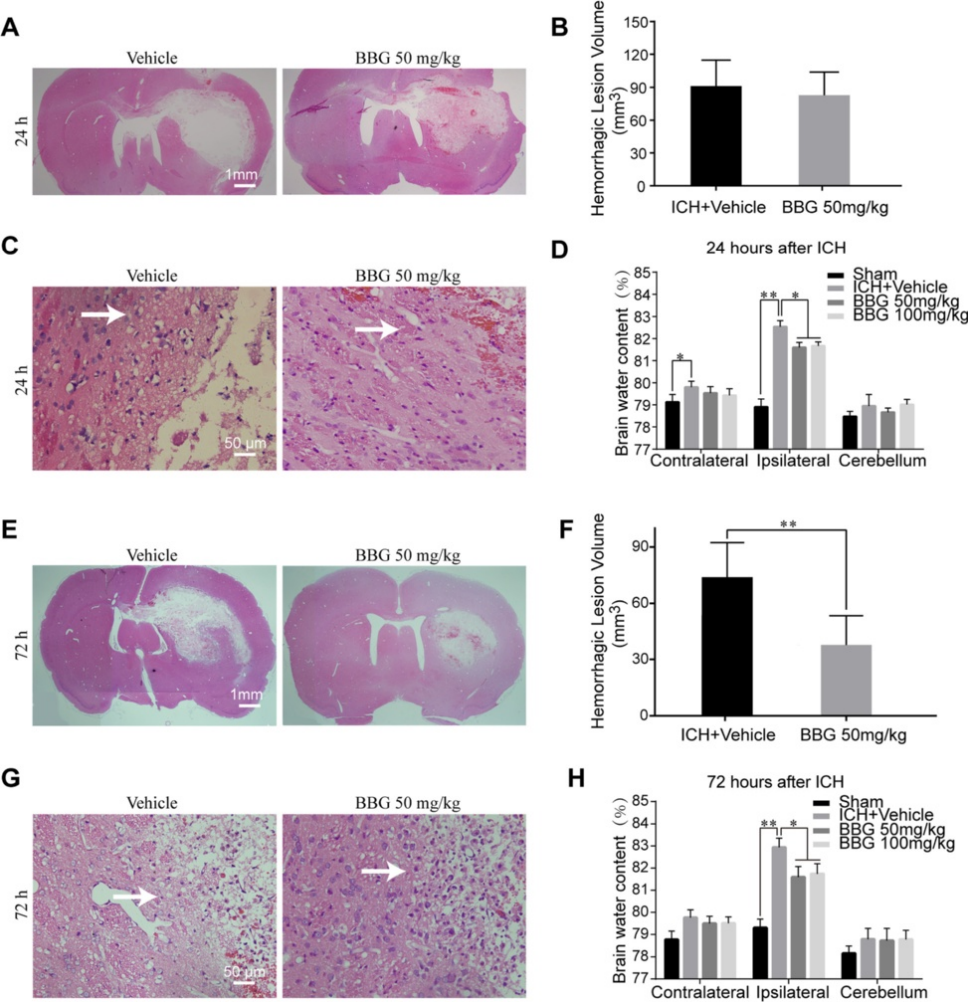
Effects of BBG on brain edema in ICH rats. BBG treatment evidently reduced brain edema at 24 (**d**) and at 72 h (**h**) after ICH. Hematoxylin and eosin (H&E) showed the hemorrhagic lesion volume alteration after BBG treatment at 24 (**a**, **b**) and at 72 h (**e**, **f**) following ICH. Tissue damage after BBG treatment at 24 (**c**) and at 72 h (**g**) following ICH. *n* = 6 rats per group. Data represent means ± SD. ^*^ *P* < 0.05, ^**^ *P* < 0.01. *BBG* brilliant blue G, *ICH* intracerebral hemorrhage

**Fig. 6:**
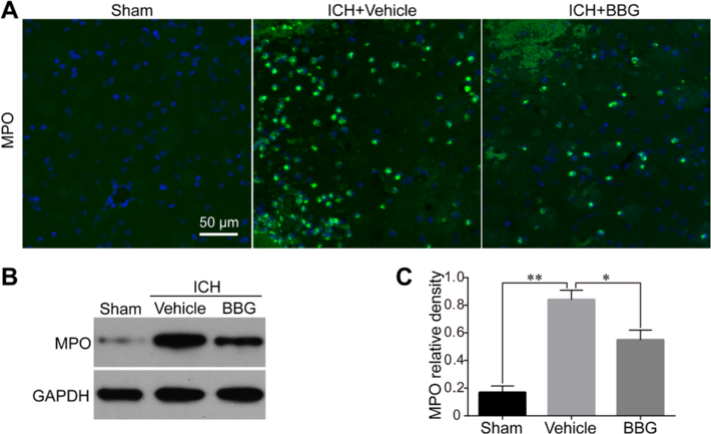
Effects of BBG on neuronal apoptosis and neurological outcomes in ICH rats. BBG significantly reduced the number of apoptotic neurons (**a**, **b**) 24 h following ICH, *n* = 6 rats per group. BBG significantly improved neurological deficits (**c**) at 24 and at 72 h after ICH, *n* = 6 rats per group. Scale bar = 50 μm. Data represent means ± SD. ^*^ *P* < 0.05, ^**^ *P* < 0.01. *BBG* brilliant blue G

Next, we investigated the effects of the selective P2X7R inhibitor, BBG. Both doses (50 and 100 mg/kg) significantly attenuated brain water content at 24 h (50 mg/kg, 81.76 ± 0.32 % vs. Vehicle, 82.54 ± 0.66 %, *P* < 0.05; 100 mg/kg, 81.67 ± 0.43 % vs. Vehicle, 82.54 ± 0.66 %, *P* < 0.05) and 72 h (50 mg/kg, 81.59 ± 1.15 % vs. Vehicle, 82.94 ± 1.00 %, *P* < 0.05; 100 mg/kg, 81.74 ± 1.12 % vs. Vehicle, 82.94 ± 1.00 %, *P* < 0.05) after ICH (Fig. 5d, h). The hemorrhagic lesion volume at 24 h post-ICH for the Vehicle and BBG groups were 91.17 ± 23.54 and 82.77 ± 21.31, respectively (*P* > 0.05) (Fig. 5a, b), indicating that BBG did not affect bleeding. However, the hemorrhagic lesion volume for the BBG group (37.79 + 15.56) was significantly decreased compared with the Vehicle (73.03 + 19.34) group at 72 h post-ICH (*P* < 0.01) (Fig. 5e, f), indicating that BBG could promote tissue reconstruction. Meanwhile, the tissue damage around the lesion site was evidently mitigated by BBG treatment at both 24 and 72 h (Fig. 5c, d, g, h). Consistently, neurological deficits (Fig. 6c) at both 24 h (50 mg/kg, 8.83 ± 1.64 vs. Vehicle, 10.00 ± 1.95, *P* < 0.05; 100 mg/kg, 8.66 ± 1.55 vs. Vehicle, 10.00 ± 1.95, *P* < 0.05) and 72 h (50 mg/kg, 6.40 ± 1.64 vs. Vehicle, 8.10 ± 1.96, *P* < 0.05; 100 mg/kg, 7.00 ± 1.78 vs. Vehicle, 8.10 ± 1.96, *P* < 0.05) after ICH were improved by BBG treatment. However, there was no difference between animals that received 50 and 100 mg/kg doses of BBG treatment with regard to mNSS scores or brain water contents. Thus, the 50 mg/kg dose was applied in further studies.

The number of apoptotic neurons was significantly increased at 24 h after ICH compared with the Sham group (*P* < 0.01), and BBG treatment significantly reduced the number of apoptotic neurons (*P* < 0.01) relative to the Vehicle group (Fig. 6a, b).

### BBG decreased P2X7R expression, NLRP3/ASC/caspase-1 activation, and subsequent IL-1β/IL-18 production following ICH

**Fig. 7:**
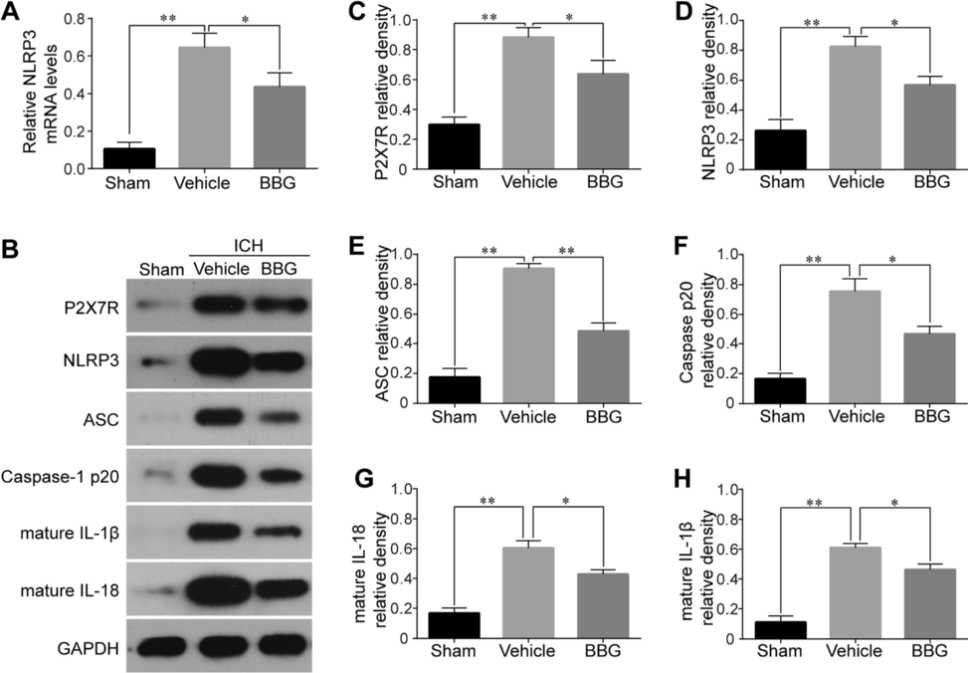
Effects of BBG on NLRP3 inflammasome activation and IL-1β/IL-18 maturation. RT-PCR of NLRP3 after BBG treatment 24 h following ICH, *n* = 6 rats per group (**a**). Representative western blot (**b**) and therapeutic effects of BBG on P2X7R (**c**), NLRP3 (**d**), ASC (**e**), caspase-1 p20 subunit (**f**), mature IL-1β (**g**), and mature IL-18 (**h**) levels in the ipsilateral hemisphere 24 h after ICH, *n* = 4 rats per group. Data represent means ± SD. ^*^ *P* < 0.05, ^**^ *P* < 0.01. *ASC* adaptor protein apoptosis-associated speck-like protein containing a CARD, *BBG* brilliant blue G, *GAPDH* glyceraldehyde 3-phosphate dehydrogenase, *ICH* intracerebral hemorrhage, *IL* interleukin, *NLRP3* pyrin domain-containing 3

BBG treatment significantly reduced NLRP3 mRNA levels (Fig. 7a). Western blot analysis revealed that BBG (50 mg/kg) treatment attenuated the expressions of P2X7R (*P* < 0.05 vs. Vehicle), NLRP3 (*P* < 0.05 vs. Vehicle), ASC (*P* < 0.01 vs. Vehicle), and cleaved caspase-1 (*P* < 0.05 vs. Vehicle). Furthermore, the levels of mature IL-1β (*P* < 0.05 vs. Vehicle) and IL-18 (*P* < 0.05 vs. Vehicle) were distinctly reduced after BBG treatment (Fig. 7b–h).

### BBG reduced neutrophils infiltration after ICH.

**Fig. 8:**
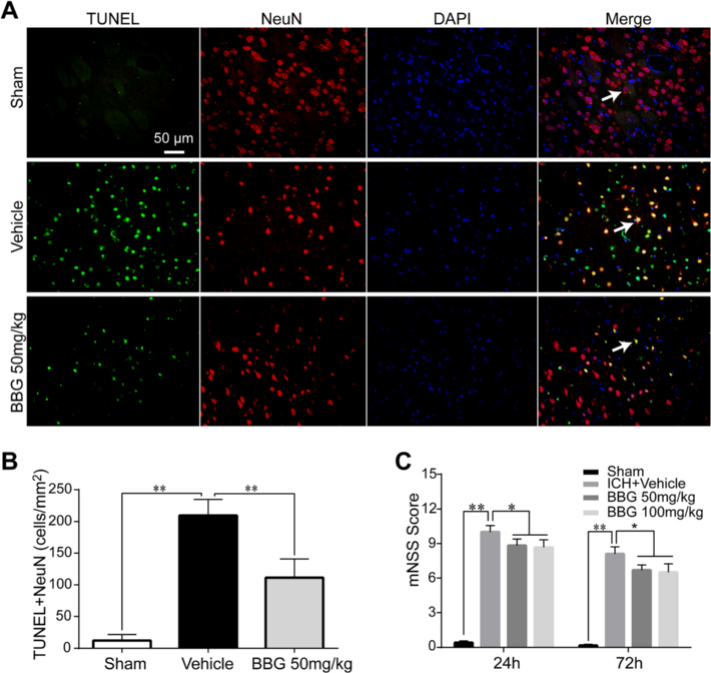
Effects of BBG on neutrophils infiltration after ICH. Representative photographs of immunofluorescence staining (**a**) for MPO (neutrophil marker)-positive cells in perihematomal area in the Sham, Vehicle, and BBG (50 mg/kg) groups at 24 h following operation, *n* = 6 rats per group. Representative western blot (**b**) and effects of BBG on MPO levels (**c**) at 24 h after ICH, *n* = 4 rats per group. Scale bar = 50 μm. Data represent means ± SD. ^*^ *P* < 0.05, ^**^ *P* < 0.01. *BBG* brilliant blue G, *ICH* intracerebral hemorrhage

We detected MPO levels in brain tissue by IF (Fig. 8a) and western blot (Fig. 8b, c) at 24 h following ICH to determine the effect of P2X7R/NLRP3 inflammasome axis activation on neutrophil infiltration. Striatal MPO levels were evidently increased following ICH compared with the Sham group (*P* < 0.01 vs. Sham). BBG (50 mg/kg) significantly suppressed MPO expression compared to the Vehicle group (*P* < 0.05 vs. Vehicle).

### BBG suppressed ICH-induced iNOS expression

**Fig. 9:**
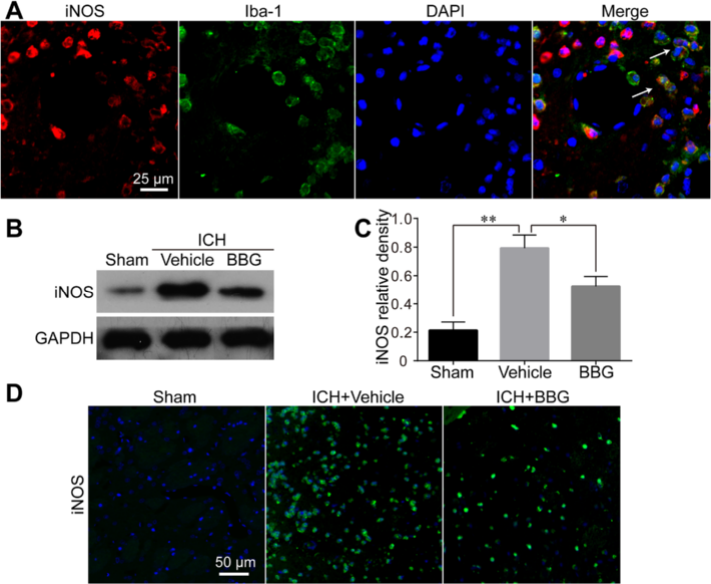
Effects of BBG on ICH-induced iNOS expression in the striatum. Most iNOS signals overlapped with Iba-1 positive microglia (**a**). Western blot (**b**, **c**) and immunofluorescence labeling (**d**) showing that BBG treatment significantly decreased striatal iNOS expression 24 h after ICH compared with the Vehicle group. *n* = 4 (**b**, **c**) or *n* = 6 (**a**, **d**) rats per group. Scale bar = 25 μm (**a**) or 50 μm (**d**). Data represent means ± SD. ^*^ *P* < 0.05, ^**^ *P* < 0.01. *BBG* brilliant blue G, *GAPDH* glyceraldehyde 3-phosphate dehydrogenase, *ICH* intracerebral hemorrhage, *iNOS* inducible nitric oxide synthase

iNOS is upregulated in both blood infusion and collagenase-induced ICH rat models [31, 35]. The role of P2X7R in iNOS signaling was next investigated using IF and western blot analysis. iNOS expression was weak in Sham-operated rats but was dramatically elevated 24 h after ICH induction (*P* < 0.01 vs. Sham). To further trace the source of iNOS, double IF was performed and found that iNOS was mainly expressed in Iba-1-positive microglia (Fig. 9a). Western blots and IF showed that the enhanced iNOS levels were markedly attenuated in BBG-treated rats (*P* < 0.05 vs. Vehicle) (Fig. 9b–d).

### BBG reduced ICH-induced NOX2 expression

**Fig. 10:**
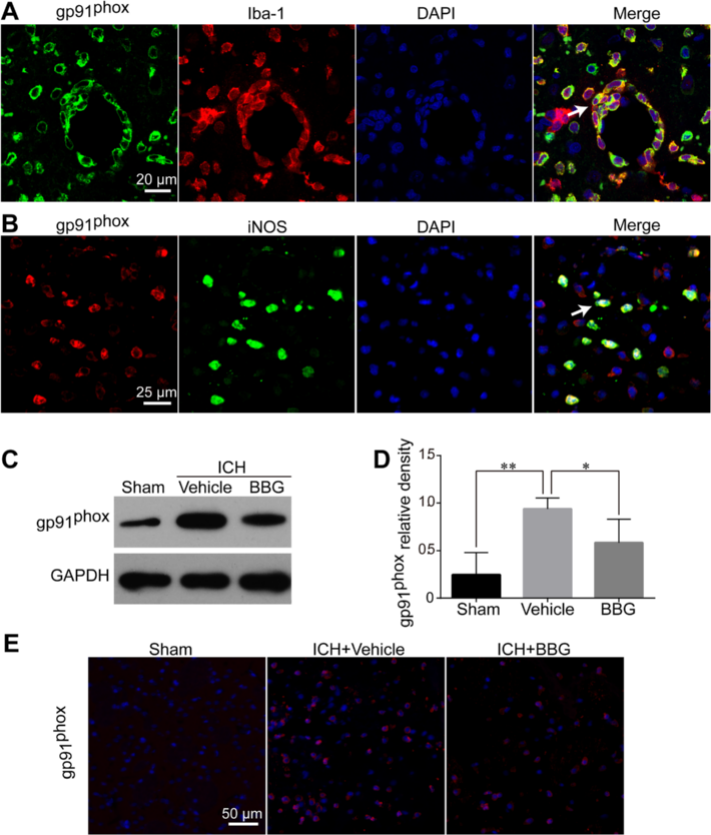
Effects of BBG on ICH-induced gp91^phox^ expression in the striatum. Gp91^phox^ significantly colocalized with Iba-1 (**a**). Most gp91^phox^ signals overlapped with iNOS (**b**). Western blot (**c**, **d**) and immunofluorescence labeling (**e**) showing that BBG treatment significantly reduced the striatal gp91^phox^ expression 24 h after ICH compared with the Vehicle group. *n* = 4 (**d**) or *n* = 6 (**e**) rats per group. Scale bar = 20 μm (**a**) or 25 μm (**b**) or 50 μm (**e**). Data represent means ± SD. ^*^ *P* < 0.05; ^**^ *P* < 0.01. *BBG* brilliant blue G, *GAPDH* glyceraldehyde 3-phosphate dehydrogenase, *ICH* intracerebral hemorrhage, *iNOS* inducible nitric oxide synthase

NOX2, a primary source of O_2_^−^, is actively involved in ICH-induced brain injury [15]. We studied the expression of gp91^phox^, a membrane subunit of NOX2, by IF and western blotting. Double IF showed that gp91^phox^ was also mainly expressed in Iba-1-positive areas (Fig. 10a) and most overlapped with iNOS expression (Fig. 10b), implying a close connection between them. Consistent with iNOS, gp91^phox^ was significantly increased after ICH in ipsilateral hemisphere brain tissues as compared with the Sham group (*P* < 0.01 vs. Sham) at 24 h after ICH. BBG treatment significantly downregulated gp91^phox^ overexpression (*P* < 0.05 vs. Vehicle) (Fig. 10c–e).

### BBG attenuated peroxynitrite formation following ICH

**Fig. 11:**
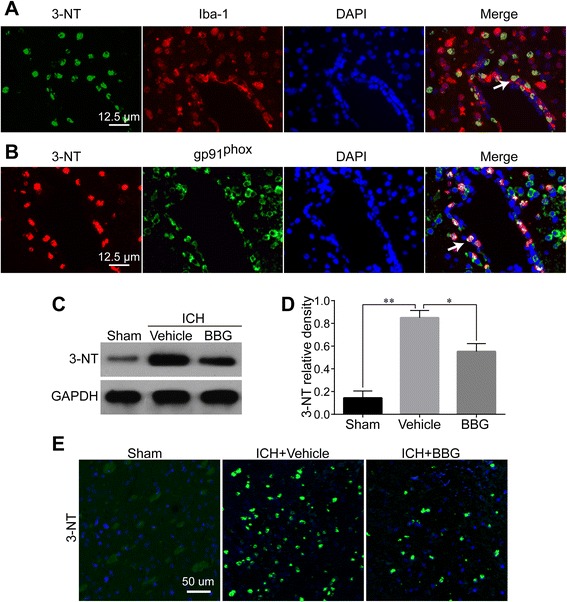
Effects of BBG on ICH-induced 3-NT expression in the striatum. 3-NT significantly colocalized with Iba-1 (**a**). Most 3-NT signals overlapped with gp91^phox^ (**b**). Western blot (**c**, **d**) and immunofluorescence staining (**e**) showing that BBG treatment significantly suppressed striatal 3-NT expression 24 h after ICH compared with the Vehicle group. *n* = 4 (**a**, **b**, **e**) or *n* = 6 (**c**, **d**) rats per group. Bar = 12.5 μm (**a**, **b**) or 50 μm (**e**). Data represent means ± SD. ^*^ *P* < 0.05, ^**^ *P* < 0.01. *BBG* brilliant blue G, *GAPDH* glyceraldehyde 3-phosphate dehydrogenase, *ICH* intracerebral hemorrhage, *3-NT* 3-nitrotyrosine

The enhancement of iNOS and NOX2 after ICH induction prompted us to examine the involvement of P2X7R in ONOO^−^ formation. Double IF demonstrated a high degree of colocalization with Iba-1 (Fig. 11a). Moreover, 3-NT and gp91^phox^ expressions were almost completely overlapped (Fig. 11b), implying that peroxynitrite production is NOX2 dependent. However, BBG treatment significantly downregulated 3-NT overexpression (*P* < 0.05 vs. Vehicle) (Fig. 11c–e).

### The ONOO^−^ decomposition catalyst FeTPPS inhibited NLRP3/ASC/Caspase-1 activation and subsequent production of mature IL-1β/IL-18 following ICH

**Fig. 12:**
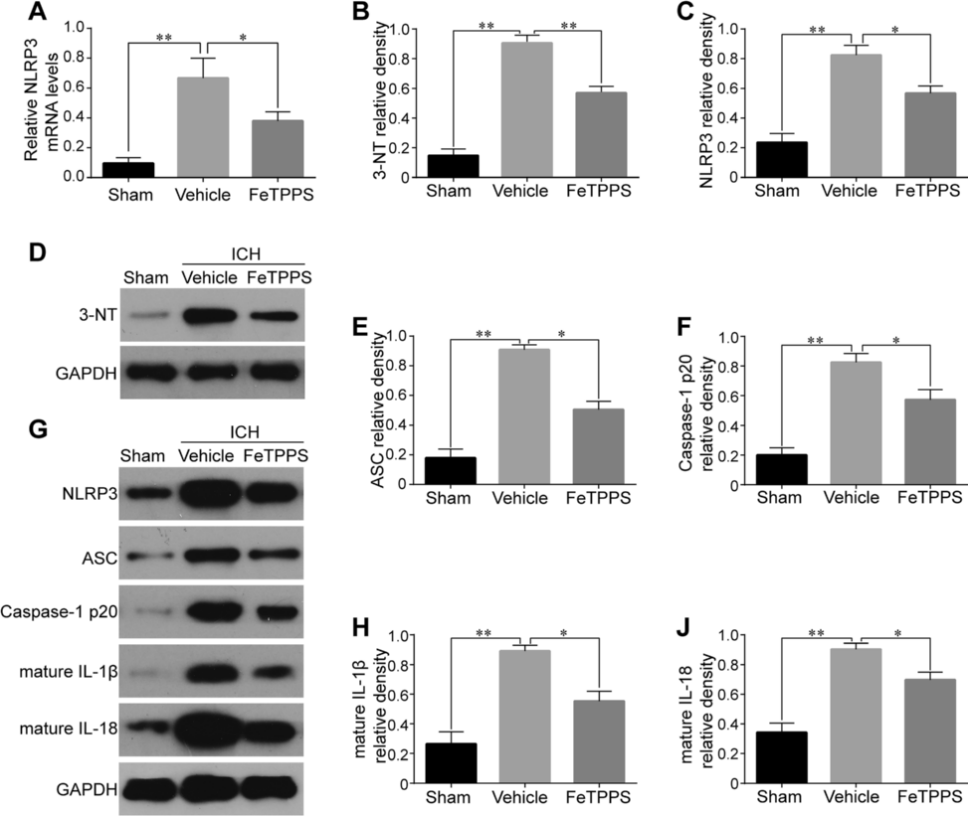
Effects of FeTPPS on NLRP3 inflammasome activation and IL-1β/IL-18 maturation. RT-PCR of NLRP3 after FeTPPS treatment 24 h following ICH, *n* = 6 rats per group (**a**). Western blot (**d**, **b**) showed that FeTPPS significantly reduced striatal 3-NT expression 24 h after ICH compared with the Vehicle group. Western blot assay (**g**) and quantification of NLRP3 (**c**), ASC (**e**), caspase-1 p20 subunit (**f**), mature IL-1β (**h**), and mature IL-18 (**j**) after FeTPPS treatment 24 h following ICH, *n* = 5 rats per group. Data represent means ± SD. ^*^ *P* < 0.05, ^**^ *P* < 0.01. *ASC* adaptor protein apoptosis-associated speck-like protein containing a CARD, *GAPDH* glyceraldehyde 3-phosphate dehydrogenase, *ICH* intracerebral hemorrhage, *IL* interleukin, *NLRP3* pyrin domain-containing 3, *3-NT* 3-nitrotyrosine

Our findings suggested a pivotal role for microglia-expressed P2X7R in mediating ONOO^−^ formation in an iNOS and NOX2-dependent way, which further prompted us to explore whether ONOO^−^ served as the key bridge between P2X7R and NLRP3 inflammasome activation. To answer this question, the ONOO^−^ decomposition catalyst FeTPPS was applied in vivo. Firstly, FeTPPS significantly reduced 3-NT levels (*P* < 0.01 vs. Vehicle) on western blot (Fig. 12a, b). Thereafter, the expression levels of P2X7R and NLRP3 inflammasome components were measured by western blot at 24 h following ICH. The results indicated that FeTPPS significantly downregulated the enhanced levels of NLRP3 (*P* < 0.05 vs. Vehicle), ASC (*P* < 0.05 vs. Vehicle), and cleaved caspase-1 (*P* < 0.05 vs. Vehicle) after ICH. Moreover, the upregulation of IL-1β/IL-18 was also attenuated by FeTPPS (*P* < 0.05 vs. Vehicle) (Fig. 12c–h). However, FeTPPS had no influence on P2X7R expressions (Additional file 3). FeTPPS treatment significantly reduced NLRP3 mRNA expression. Together, these results reveal that P2X7R-dependent synthesis of ONOO^−^ may be a key activator of the NLRP3 inflammasome.

## Discussion

Innate immune and inflammatory responses are increasingly recognized as important factors in the pathophysiology of secondary brain injury following ICH. The NLRP3 inflammasome, the most characterized pattern recognition receptor (PRR) in innate immune response initiation, is strongly involved in ICH-induced inflammation and produces pro-inflammatory factors such as IL-1β [2].

Several distinct mechanisms have been proposed to account for NLRP3 activation, including potassium (K^+^) efflux, intracellular calcium alteration, ubiquitination, and reactive oxygen species (ROS) generation [36]. It was recently reported that extracellular ATP-induced P2X7R activation could directly mediate K^+^ efflux through the hemichannel pannexin 1 to activate the NLRP3 inflammasome [5, 28, 36]. However, pannexin 1-deficient mice do not show diminished caspase-1 activation [37], implying that pannexin-1 is dispensable for the assembly of caspase-1 activating inflammasome complexes. This raises the hypothesis that other signaling pathways may be involved in P2X7R-mediated NLRP3 inflammasome activation or that something besides pannexin 1 is necessary for P2X7R-dependent K^+^ efflux. NOX2-derived ROS after ATP release-mediated P2X7R activation is well established in a growing body of in vitro and in vivo disease models [23–25]. Importantly, there are a number of reports supporting that high ROS levels, particularly NOX2-derived ROS, are critical for NLRP3 inflammasome activation [36]. RNS also play an important role as evidenced by the fact that both an ONOO^−^ scavenger and NOX2 inhibitor suppressed nigericin-induced caspase-1 activation and IL-1β secretion in human monocytes [28]. Additionally, K^+^ efflux can be positively regulated by ROS [14] and RNS (e.g., ONOO^−^) [38]. In this regard, we propose that NOX2-mediated ONOO^−^ formation may link P2X7R and NLRP3 inflammasome activation.

P2X7R activation in the ICH brain is a novel finding of this study. ATP is released in large quantities following any kind of cell injury [39], and cell death occurs 3 to 6 h after collagenase- induced ICH [40]. Elevated extracellular ATP is necessary for P2X7R activation [13]. In the present study, increased P2X7R was observed as early as 6 h after ICH in the perihematomal tissue along with expression of the microglia marker Iba-1, suggesting that P2X7R may act as a signal in the process of microglia activation as previously reported [41].

Microglia are believed to be one of the first non-neuronal cells to respond to brain injury [42], much earlier than neutrophil invasion [43]. Activated microglia then release pro-inflammatory cytokines, such as IL-1β [44] and IL-18 [45], which recruit leukocytes especially neutrophils. The infiltrated neutrophils amplify neuroinflammation by releasing and expressing neurotoxic factors or even by stimulating microglia to secrete neurotoxic factors, creating a vicious cycle [46]. In the present study, upregulated P2X7R was closely associated with increased NLRP3/ASC/caspase-1 inflammasome expression; siRNA interference or pharmacological blockage of P2X7R impaired inflammasome activation and IL-1β/IL-18 secretion, which had a pronounced neuroprotective effect. Moreover, infiltrated neutrophils were also diminished in the BBG group compared with the Vehicle group. These results indicate that P2X7R may be responsible for ICH-induced inflammation, possibly by NLRP3 inflammasome-dependent IL-1β/IL-18 release and subsequent neutrophil infiltration signals. In accordance with our results, Ma and colleagues [2] found that NLRP3 mainly colocalized with microglia but not with other cell types, providing further support for a tight relationship between P2X7R and NLRP3.

In response to microglia activation, large amounts O_2_^−^ and NO are released through activated NADPH oxidase and iNOS [17], both of which play pivotal roles in ICH-induced brain damage [15, 16]. In our study, enhanced gp91^phox^ in microglia largely colocalized with iNOS, prompting us to consider ONOO^−^. Double IF revealed that increased 3-NT was also expressed in microglia and mostly overlapped with gp91^phox^. Consistently with this, Mander and Brown [47] reported that activation of NOX or iNOS alone was relatively harmless, but their simultaneous activation was lethal because it spurred ONOO^−^ production. Inhibition of either iNOS [48] or NOX2 [49] significantly reduced ONOO^−^ production in CNS disease models. Collectively, we can infer that NOX2-derived O_2_^−^ and iNOS-derived NO may contribute to ONOO^−^ formation upon microglia activation after ICH.

We also found that ICH-induced upregulations of NOX2, iNOS, and ONOO^−^ were diminished in BBG treated ICH rats, indicating that they may be downstream of P2X7R as previously reported [26, 29]. We next explored the role of ONOO^−^ in NLRP3 inflammasome activation. As a highly active and relatively specific ONOO^−^ decomposition catalyst, FeTPPS exerts neuroprotective effects in many CNS disease models [20, 50] because it catalyzes peroxynitrite to become a harmless nitrate [51]. Our results show that overexpression of NLRP3 inflammasome components and mature IL-1β/IL-18 was reduced by FeTPPS. These findings suggest that ONOO^−^ may be involved in inflammasome activation following ICH. Consistent with our results, an early report found that FeTPPS inhibited nigericin-induced caspase-1 activation and IL-1β secretion in human monocytes [28].

Although we did not attempt to explore the exact mechanisms of ONOO^−^ in modulating NLRP3 inflammasome activation, ONOO^−^ may act as a key mediator for inflammasome activation through several mechanisms. Firstly, direct oxidation of mitochondria and the release of mitochondrial DNA (mtDNA) activate the NLRP3 inflammasome; a recent study showed that oxidized mtDNA released into the cytosol from injured mitochondria could bind to and activate the NLRP3 inflammasome [52]. ONOO^−^ is a potent ROS with strong abilities to oxidize and nitrate proteins, lipids, and DNA. Mitochondria are a primary target for peroxynitrite [18]. Secondly, nitration can inactivate thioredoxin (Trx), leading to the dissociation of thioredoxin interaction protein (TXNIP) from Trx. Zhou et al. [53] reported that TXNIP could dissociate from Trx in an ROS-sensitive way, allowing it to bind and activate NLRP3. Thirdly, ONOO^−^ may mediate K^+^ efflux and then activate NLRP3. K^+^ efflux is a well-characterized activator for NLRP3 inflammasome, and ONOO^−^ is a positive modulator for K^+^ efflux [38]. Therefore, ONOO^−^ may be responsible for NLRP3 inflammasome activation.

Of translational significance, we investigated the effect of BBG in an ICH rat model. Previous studies have demonstrated a neuroprotective effect of BBG in many acute CNS diseases such as ischemic stroke [54], subarachnoid hemorrhage [6], traumatic brain injury [55], and spinal cord injury [56]. BBG is a derivative of the widely used and Food and Drug Administration-approved food additive FD&C Blue number 1 [57]. With its low toxicity and high selectivity, BBG is considered to be an attractive drug for CNS diseases [56]. We found that BBG treatment inhibited the inflammatory response via the P2X7R/NLRP3 axis following ICH, and this was associated with significantly improved neurological function and less brain edema.

Several potential limitations deserve mention. Firstly, although we employed the most commonly used ICH model and injected sterile-filtered collagenase, the extra-inflammatory responses induced by the collagenase itself seemed unavoidable. Secondly, our observation period was 72 h but it appears that ASC and caspase-1 activation may undergo a second peak at 72 h; we speculate that other signaling pathways may be involved in ASC and caspase-1 activation in a later phase. Finally, although it was significant, the effect of P2X7R siRNA and BBG on changes in brain water content and neurobehavioral scores was relatively small. Thus, further experiments are necessary to address these problems.

## Conclusion

In summary, our results indicate that P2X7R contributes to NLRP3 inflammasome activation and subsequent IL-1β/IL-18 release to drive brain inflammation and neuronal damage in an ICH rat model. NOX2/iNOS-dependent ONOO^−^ formation, a potential downstream signaling component of P2X7R, may be a key trigger of NLRP3 inflammasome activation. Thus, inhibition of P2X7R or ONOO^−^ could be a potential therapeutic target for secondary brain injury accompanying ICH.

## Additional files

Additional file 1:
**Experiment design and animal group classification.** ICH = intracerebral hemorrhage; WB = western blotting; BWC = brain water content; IF = immunofluorescence; BBG = brilliant blue G. (TIFF 891 kb) Additional file 2:
**No evident immunostaining for P2X7R was found in astrocytes and neurons.** Double immunostaining showed that P2X7R was not expressed in GFAP positive astrocytes (A). Double immunostaining showed that P2X7R was not expressed in NeuN positive neurons (B). (TIFF 4781 kb) Additional file 3:
**FeTPPS treatment had no influence on P2X7R expressions.** Western blot (A,B) showed that FeTPPS did not affect the protein expressions of P2X7R. ^***^ *P* < 0.05, ^****^ *P* < 0.01 (TIFF 494 kb)

## Abbreviations

3-NT: 3-nitrotyrosine
ASC: apoptosis-associated speck-like protein containing a CARD
ATP: adenosine triphosphate
BBG: brilliant blue G
CNS: central nervous system
ICH: intracerebral hemorrhage
IL: interleukin
iNOS: inducible nitric oxide synthase
LPS: lipopolysaccharide
mNSS: modified Neurological Severity Score
MPO: myeloperoxidase
mtDNA: mitochondrial DNA
NLRP3: NLR family, pyrin domain-containing 3
NO: nitric oxide
NOX2: NADPH oxidase 2
O_2_^−^: superoxide anion
ONOO^−^: peroxynitrite
P2X7R: purinergic 2X7 receptor
RNS: reactive nitrogen species
ROS: reactive oxygen species
SD: Sprague–Dawley
Sham: sham-operated animals
siRNA: small interfering RNA
TUNEL: terminal deoxynucleotidyl transferase-mediated dUTP nick 3′-end labeling
