# Erratum to: P2X7R blockade prevents NLRP3 inflammasome activation and brain injury in a rat model of intracerebral hemorrhage: involvement of peroxynitrite

**DOI:** 10.1186/s12974-016-0627-2

**Published:** 2016-08-17

**Authors:** Liang Feng, Yizhao Chen, Rui Ding, Zhenghao Fu, Shuo Yang, Xinqing Deng, Jun Zeng

**Affiliations:** 1 The National Key Clinical Specialty, The Engineering Technology Research Center of Education Ministry of China, Guangdong Provincial Key Laboratory on Brain Function Repair and Regeneration, Department of Neurosurgery, Zhujiang Hospital, Southern Medical University, Guangzhou, 510282 China; 2 Department of Neurosurgery, Jingmen No. 1 People’s Hospital, Jingmen, Hubei 448000 China; 3 Department of Neurosurgery, The Fifth Affiliated Hospital of Southern Medical University, Guangzhou, 510900 China; 4 Department of Neurosurgery, Gaoqing Campus of Central Hospital of Zibo, Gaoqing People’s Hospital, Gaoqing, Zibo, Shandong 256300 China; 5 Department of Neurosurgery, 999 Brain Hospital, Jinan University, Guangzhou, Guangdong 510510 China

## Erratum

After publication of this work [1], it was noted that there was an error within Figs. 6 and 8: both these figures were inadvertently transposed with one another. This has been correctly updated in the original article, and is also included correctly below.

**Fig. 6:**
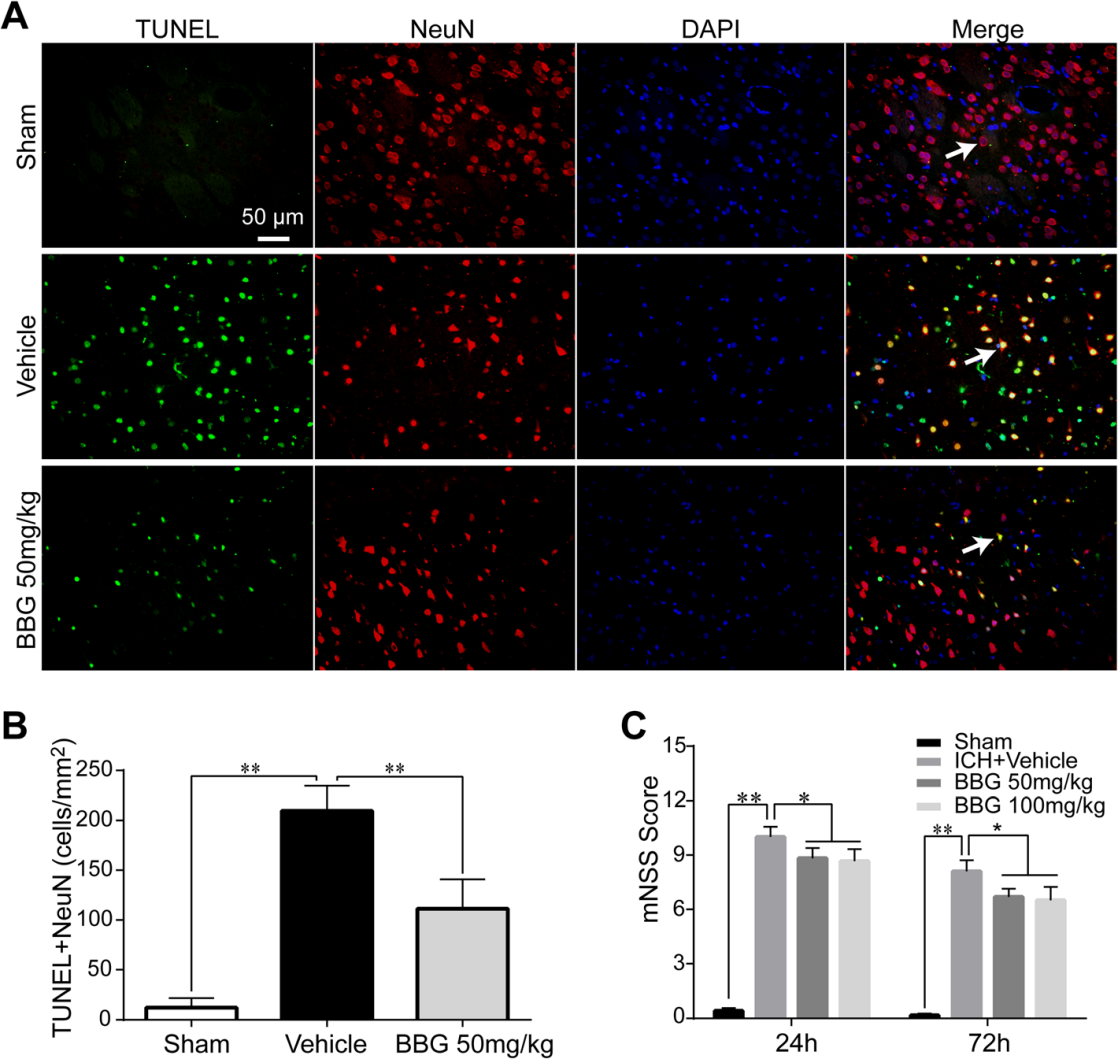
Effects of BBG on neuronal apoptosis and neurological outcomes in ICH rats. BBG significantly reduced the number of apoptotic neurons (**a**, **b**) 24 h following ICH, n = 6 rats per group. BBG significantly improved neurological deficits (**c**) at 24 and at 72 h after ICH, n = 6 rats per group. Scale bar = 50 μm. Data represent means ± SD. * P < 0.05, ** P < 0.01. BBG brilliant blue G

**Fig. 8:**
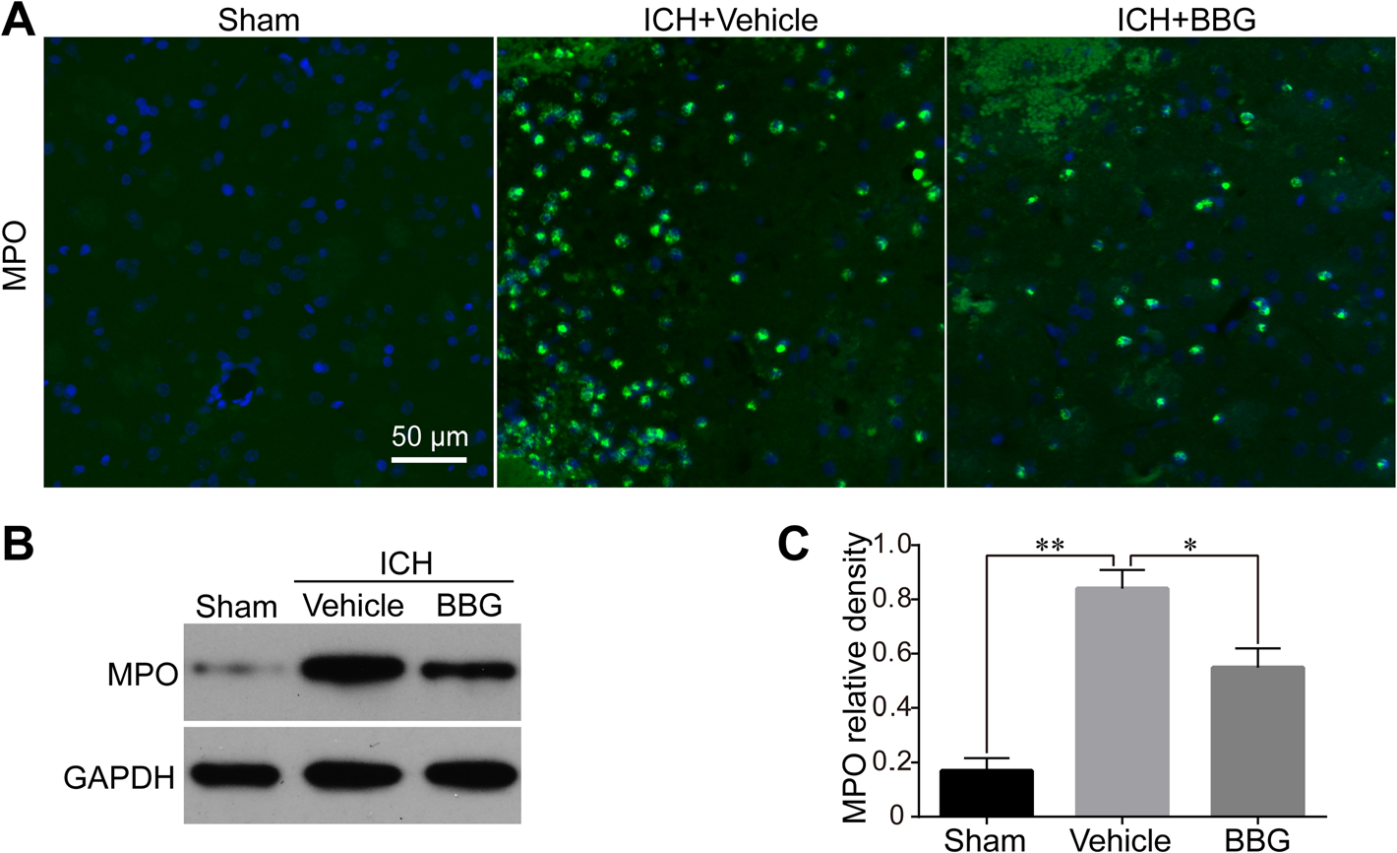
Effects of BBG on neutrophils infiltration after ICH. Representative photographs of immunofluorescence staining (**a**) for MPO (neutrophil marker)-positive cells in perihematomal area in the Sham, Vehicle, and BBG (50 mg/kg) groups at 24 h following operation, n = 6 rats per group. Representative western blot (**b**) and effects of BBG on MPO levels (**c**) at 24 h after ICH, n = 4 rats per group. Scale bar = 50 μm. Data represent means ± SD. * P < 0.05, ** P < 0.01. BBG brilliant blue G, ICH intracerebral haemorrhage

## References

[1] Feng L, Chen Y, Ding R, Fu Z, Yang S, Deng X (2015). P2X7R blockade prevents NLRP3 inflammasome activation and brain injury in a rat model of intracerebral hemorrhage: involvement of peroxynitrite. J Neuroinflammation.

