# Supplementary figures and images for: P2X7R blockade prevents NLRP3 inflammasome activation and brain injury in a rat model of intracerebral hemorrhage: involvement of peroxynitrite
Source: J Neuroinflammation. 2015 Oct 17;12:190. doi: 10.1186/s12974-015-0409-2 (PMC4609067; doi:10.1186/s12974-015-0409-2)

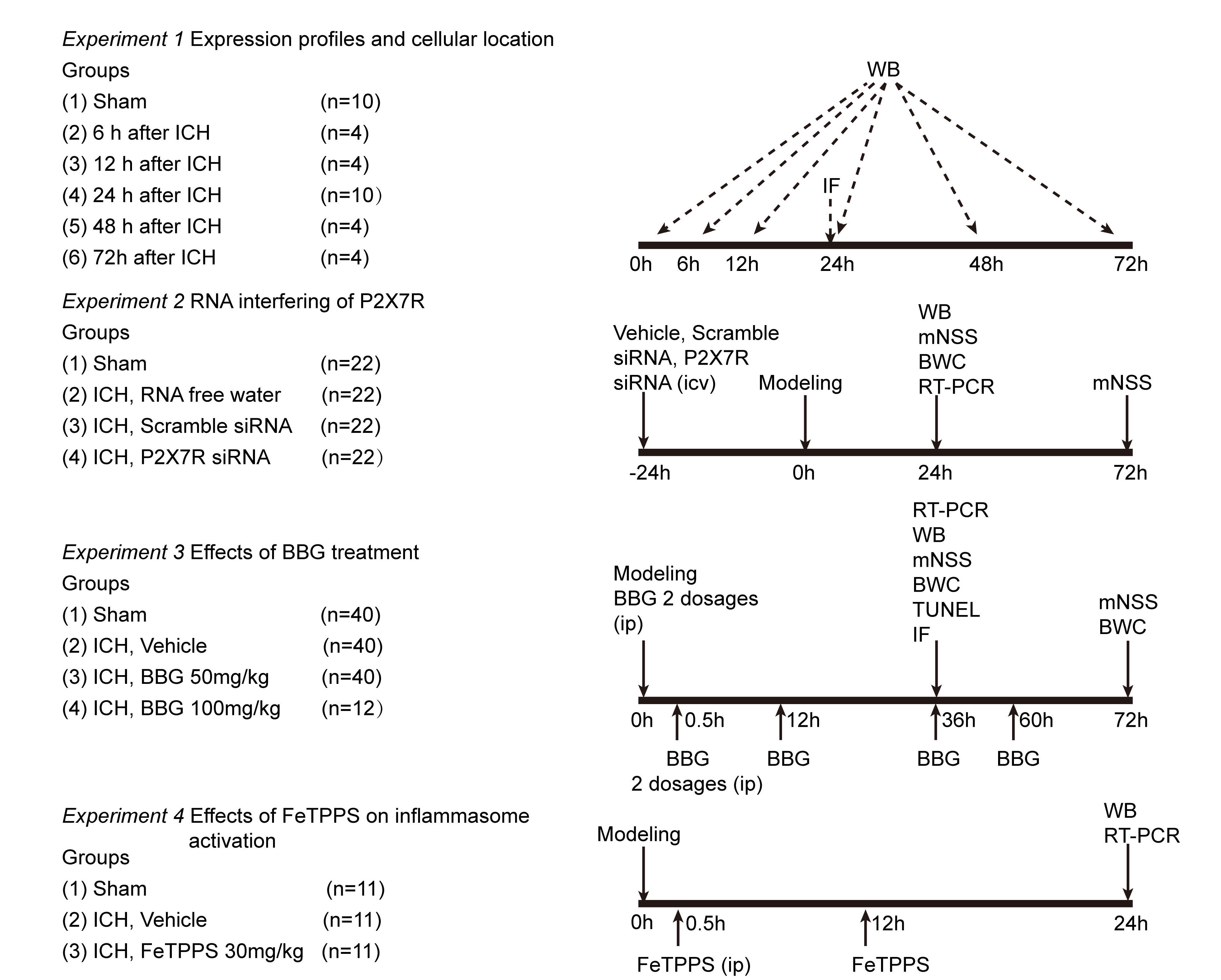

Supplement: Additional file 1: — Experiment design and animal group classification. ICH = intracerebral hemorrhage; WB = western blotting; BWC = brain water content; IF = immunofluorescence; BBG = brilliant blue G. (TIFF 891 kb) [file 12974_2015_409_MOESM1_ESM.tif]

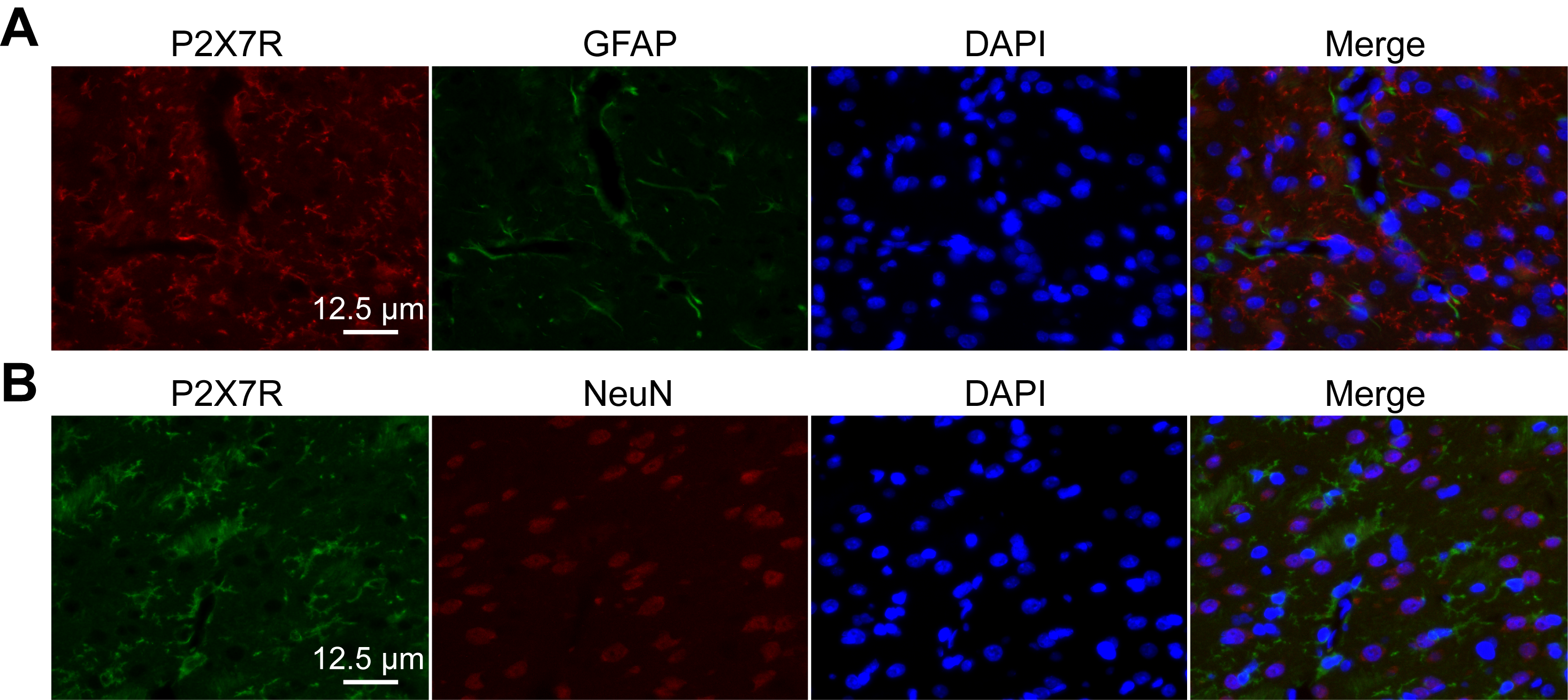

Supplement: Additional file 2: — No evident immunostaining for P2X7R was found in astrocytes and neurons. Double immunostaining showed that P2X7R was not expressed in GFAP positive astrocytes (A). Double immunostaining showed that P2X7R was not expressed in NeuN positive neurons (B). (TIFF 4781 kb) [file 12974_2015_409_MOESM2_ESM.tif]

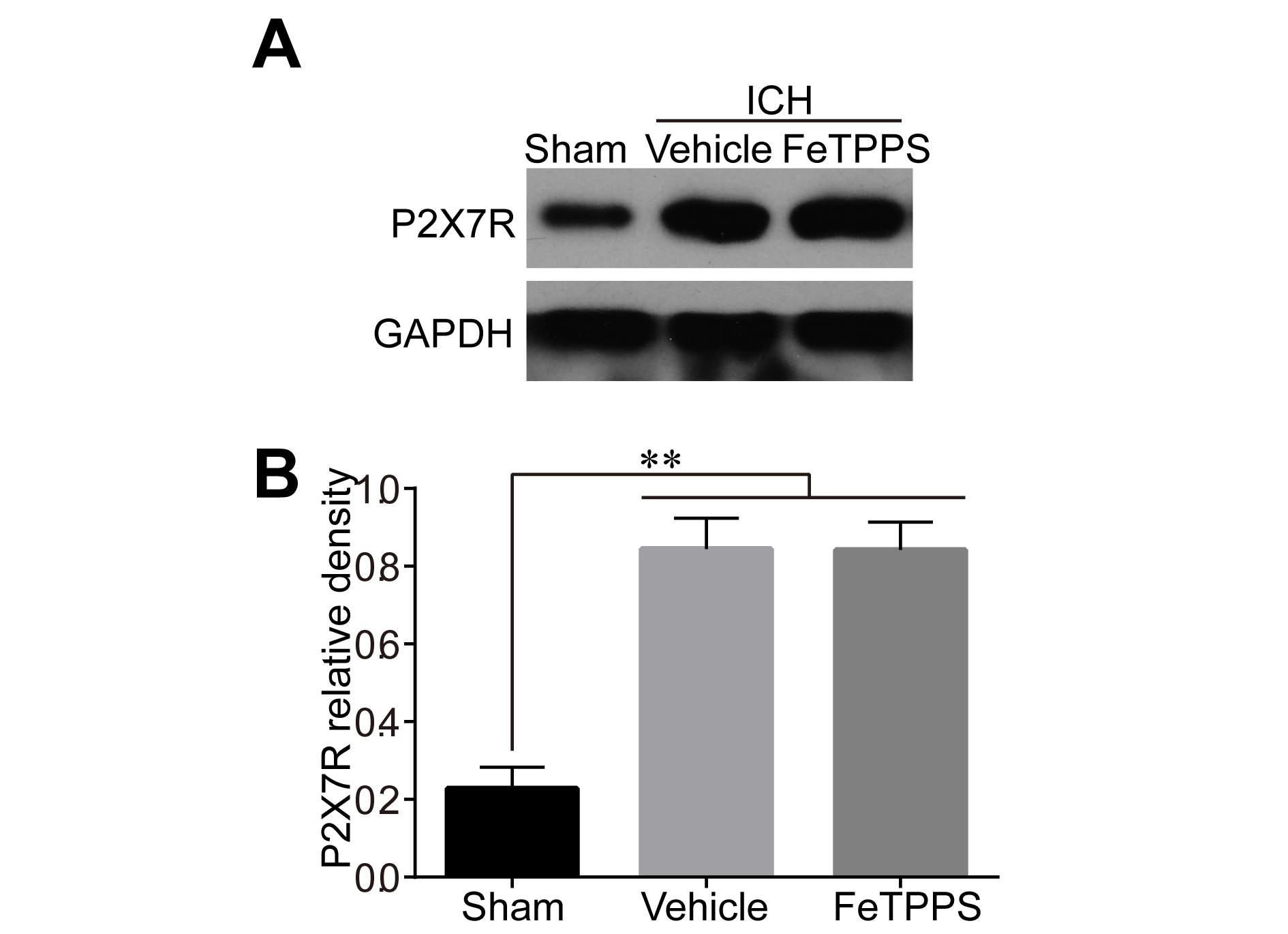

Supplement: Additional file 3: — FeTPPS treatment had no influence on P2X7R expressions. Western blot (A,B) showed that FeTPPS did not affect the protein expressions of P2X7R. * P < 0.05, ** P < 0.01 (TIFF 494 kb) [file 12974_2015_409_MOESM3_ESM.tif]
